# Whole Genome Duplication Affects Evolvability of Flowering Time in an Autotetraploid Plant

**DOI:** 10.1371/journal.pone.0044784

**Published:** 2012-09-19

**Authors:** Sara L. Martin, Brian C. Husband

**Affiliations:** Department of Integrative Biology, University of Guelph, Guelph, Ontario, Canada; North Carolina State University, United States of America

## Abstract

Whole genome duplications have occurred recurrently throughout the evolutionary history of eukaryotes. The resulting genetic and phenotypic changes can influence physiological and ecological responses to the environment; however, the impact of genome copy number on evolvability has rarely been examined experimentally. Here, we evaluate the effect of genome duplication on the ability to respond to selection for early flowering time in lines drawn from naturally occurring diploid and autotetraploid populations of the plant *Chamerion angustifolium* (fireweed). We contrast this with the result of four generations of selection on synthesized neoautotetraploids, whose genic variability is similar to diploids but genome copy number is similar to autotetraploids. In addition, we examine correlated responses to selection in all three groups. Diploid and both extant tetraploid and neoautotetraploid lines responded to selection with significant reductions in time to flowering. Evolvability, measured as realized heritability, was significantly lower in extant tetraploids (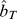 = 0.31) than diploids (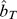 = 0.40). Neotetraploids exhibited the highest evolutionary response (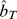 = 0.55). The rapid shift in flowering time in neotetraploids was associated with an increase in phenotypic variability across generations, but not with change in genome size or phenotypic correlations among traits. Our results suggest that whole genome duplications, without hybridization, may initially alter evolutionary rate, and that the dynamic nature of neoautopolyploids may contribute to the prevalence of polyploidy throughout eukaryotes.

## Introduction

The evolutionary significance of whole genome duplication, polyploidy, has been debated since it was discovered a century ago [1]–[7]. Biologists have primarily explored this problem by examining the phenotypic and genetic differences between polyploids and their diploid progenitors. These investigations have shown that polyploids can differ with respect to nuclear volume, organ size, developmental rate, and fertility, and that polyploids can be ecologically differentiated and reproductively isolated from their diploid progenitors (reviewed by [8]). Polyploidy is also associated with increased genetic diversity (reviewed by [9]) and species richness [10]–[13]. However, it is still unclear whether genome duplication typically increases or decreases a species' ability to respond to selection [14], [15].

Theoretical models suggest that the effect of genome duplication on the ability to respond to selection will depend on the factors that limit adaptation. When adaptive variability is limiting, genome duplication could enhance a population's ability to respond to selection by increasing the frequency of mutations and capacity for genetic diversity [6]. Alternatively, evolvability may be diminished after genome duplication if adaptation is limited by the rate of spread of a beneficial allele (i.e. the efficiency of selection) since mutations are more likely to be masked [6], [16]. The magnitude of these contradictory effects will depend on population size, mutation rate, reproductive system and the level of dominance of beneficial alleles [6]. While the effects of diploidy compared to haploidy have been explored using isogenic lines of yeast [17]–[19], there have been no experimental evaluations of natural diploid and polyploid populations, nor comparisons of extant and newly formed polyploids.

Recent research suggests allopolyploids have the potential to undergo accelerated evolution [20]. For example, work with resynthesized allopolyploids including *Brassica napus*, *Arabidopsis suecica*, *Gossypium*, *Spartina anglica*, *Hepatica*, *Nicotiana tabacum*, and *Triticum-Aegilops* has shown that phenotypic diversity may increase in new polyploid lineages due to rapid changes in gene expression, chromosomal rearrangements, retrotransposon activation, epigenetic changes, and altered genetic covariance among traits [21]–[30]. However, because allopolyploids arise through hybridization as well as genome duplication, it is not clear whether genome duplication *per se* or hybridization is responsible for increasing phenotypic diversity. Neoautopolyploids offer the opportunity to investigate the results of genome duplication without the effects of hybridization but, with a few notable exceptions [31]–[36], the phenotypic changes associated with recent autopolyploidization have received less attention than those associated with allopolyploidization [37], [38]. Importantly, the consequences of these genetic and phenotypic changes on the rate of adaptive response have not been measured directly in either autopolyploids or allopolyploids.

The species *Chamerion angustifolium* L. Holub (Onagraceae; formerly *Epilobium angustifolium;* fireweed) provides an opportunity to investigate how genome duplication alters evolvability as the species comprises diploids and autotetraploids, which were derived through genome duplication without hybridization. We assessed the effect of genome duplication on evolvability using populations of three ploidies: 1) diploids from natural populations; 2) autotetraploids from natural populations; and 3) newly synthesized autotetraploids derived from extant diploid populations, by measuring the evolutionary response to four generations of artificial selection for early flowering time. Additionally, since polyploidy may relax correlations among traits through neofunctionalization and subfunctionalization of the additional gene copies, we also assessed correlated responses to selection [39]–[44]. Specifically, we focused on examining nine reproductive and vegetative traits that may be involved in trade-offs between timing of reproduction and reproductive output following the final round of selection. These included measures of the investment in plant size (i.e. plant height at first flower), floral display (i.e. inter-style distance), plant development rate (i.e. rosette size at 3 weeks), as well as DNA content, as this effects the length of the cell cycle.

Studies of molecular variation and reciprocal transplants indicate that diploid and autotetraploid fireweed populations are genetically variable and weakly differentiated [45], [46], reflecting a large effective population size. Therefore, we anticipated that the response to selection in natural populations of diploid and autotetraploid fireweed is more likely to be limited by the efficiency of selection than the rate at which beneficial mutations arise in the population for traits that show variation. Given that diploids are expected to have the advantage when efficiency of selection limits adaptation, we predicted that for fireweed the extant polyploids should have reduced evolvability compared to diploids. Additionally, while extant autotetraploids will have accrued genetic variability through mutation and gene flow since duplication, synthesized neoautotetraploids are expected to contain a subset of the variability within the extant diploid populations. As a result, we predicted the neoautotetraploids would have the slowest response to selection as they have reduced genetic variability compared to natural autotetraploids and reduced efficiency of selection compared to diploids [21]–[30].

## Methods

### Plant material

Fireweed is a highly outcrossing, perennial herbaceous plant of open or disturbed environments in the northern hemisphere that comprises diploid (2n = 36) and tetraploid (2n = 72) individuals in mixed- and single-ploidy populations [47]–[49]. Based on morphological, cytological and genetic evidence, tetraploids are considered autotetraploids [47]. In North America, diploids and tetraploids occur at high and low latitudes/elevations respectively, with mixed populations at intermediate elevations. Flowering time is likely an important determinant of reproductive success in fireweed, as ploidies strongly sort along ecological gradients with the shorter, smaller, earlier flowering diploids typically occupying habitats with shorter growing seasons [49]. Experimental plants were derived from seed collected from diploid, tetraploid and mixed-ploidy populations within a 500 km radius in the diploid-tetraploid contact zone of the Canadian Rocky Mountains in Alberta, Canada. Specifically, diploid lines were derived from a single diploid population (Marmot Basin: 52°48.176′N, 118°04.700′W) and tetraploid lines from one tetraploid population (Fisher Creek: 50°48.449′N, 114°35.105′W). Diploid seed used to generate the neopolyploid lines was derived from a larger number of populations due to the low rate of conversion and included material from two diploid populations (Fortress Mountain: 50°49.004′N, 115°11.670′W; and Wilcox Creek: 52°27.002′N, 117°26.599′W) and four mixed-ploidy populations (Norquay 51°12.240′N , 115°35.783′W; Rampart Creek 52°02.498′N , 116°51.835′W; Coleman 50°18.540′N, 114°36.748′W; Continental Divide 51°13.673′N, 116°02.910′W). Previous studies indicate that these locations can be treated as one panmictic population due to extensive seed dispersal, high outcrossing rates, and the weak genetic differentiation between populations documented in this species [45], [50], [51]. All necessary permits were obtained from Parks Canada and Alberta's Tourism, Parks & Recreation Ministry to collect the seed used.

We synthesized neotetraploids from diploid seed by bathing approximately 30 seedlings from each of 250 diploid seed families collected from the field in a 0.02% colchicine for 18 hours. Seedlings were rinsed with distilled water and transplanted into square plug trays. Leaf tissue was sampled from each seedling at the rosette stage and screened for tetraploidy using flow cytometry. Leaf samples were hand chopped in NIB buffer [52] with leaves from a plant DNA content standard, *Epilobium hirsutum*, and stained with 50 µm ml^−1^ propidium iodide and 50 µm ml^−1^ RNase. Fluorescence data was measured (FL2 detector, 585/42 nm) using a FACSCalibur flow cytometer (BD Biosciences, Calif, USA), acquired using CellQuestPro (Benton, Dickinson and Company, 2001), and the position of the fluorescent peaks calculated using Modfit LT v3.1 (Verity Software House, ME, USA). Samples were run on two separate days to ensure an accurate assessment of DNA content [52], [53]. Tetraploid individuals were readily identified as DNA content values for tetraploids are distinct from and approximately twice that of diploids [49]. Transformed plants were transplanted into 1.5 L pots. In total, 55 fertile plants were reciprocally crossed generating 29 pairs of neotetraploid seed families. Three of the parents contributed to two different seed families.

### Selection Experiment

Tetraploid, diploid and neotetraploid seed were germinated on moist filter paper in petri dishes and incubated in a growth chamber for seven days (16 hour light cycle at 24°C). We planted seedlings from each seed family into 1.5 L pots filled with a mixture of twelve parts Sunshine Pro Planting Mix (Sun Gro Horticulture Canada) to one part MVP turface (Profile Products LLC, USA). We randomly positioned each pot in a greenhouse at the University of Guelph Phytotron and re-randomized locations three weeks later.

We established two selection lines and a control line for diploids, tetraploids, and neotetraploids. First, base populations were formed, comprising two individuals from each of 113 diploid maternal families collected in from field, 105 tetraploid maternal families collected in the field and 29 pairs of neotetraploid maternal families generated by crossing diploids that were converted to tetraploidy using colchicine (table 1).We choose to select for earlier flowering time because of the geographic distribution of cytotypes indicates the trait is ecologically relevant, because flowering time has be used in other work on autopolyploids [54], [55], and because it is practical for a selection experiment of this size. Selection early flowering time was chosen over selection for late flowering as late flowering is more likely to result from developmental abnormalities (i.e. metabolic or leaf abnormalities) than changes to the alleles that control flowering time [54]. The first 48 fertile plants (24 plants in subsequent generations) of each ploidy to flower were randomly assigned to one of two selected lines with 24 plants each with the restriction that where both maternal siblings were ranked in the first 48 the two siblings were assigned to different lines. In subsequent generations of selection lines, if more than one sibling was among the first 24 to flower, only the first sibling to flower was included in the selection group and the 25^th^ plant to flower was added to the list. This was done to reduce inbreeding as inbreeding depression is high in fireweed [48]. Control lines were established using twenty-four plants of each ploidy that were randomly chosen from the entire base population, including those assigned to selection lines, again with the restriction that multiple siblings could not be included. Plants in each line were reciprocally crossed to three non-relatives within the same line resulting in 36 pairs of seed families (pairs with the two parental plants each in the role of sire and dam). One of each of these pairs was chosen at random to contribute to the next generation and four individuals from each family formed the next generation. However, the number of families and individuals surviving until the end of the generation varied due to mortality (table 1). This protocol was repeated for four generations across all ploidies, with one exception. Although the levels of sterility among the diploids were similar to those among the neotetraploids, the small neopolyploid base population meant that few individuals (n = 41) were available for selection and, as a result, 20 plants were selected for a single selected line and 20 plants were chosen at random for a control line. These lines shared 12 individuals. Plants from both the selection and control lines were reciprocally crossed to four non-relatives resulting in 40 pairs of seed families. To establish the next generation, 36 seed family pairs were chosen with replacement at random to form each of the two neotetraploid lines. In each case, only 30 maternal groups survived (table 1). The subsequent neopolyploid generations were crossed and selected as with the diploid and tetraploid lines. The last generation was also a special case for all ploidies as we grew individuals from each generation simultaneously in a common environment to provide a second check on changes in flowering time. As a result, fewer families and individuals (two/family) were reared per line for the final generation of plants (table 1).

**Table 1:** Number of individuals involved in each stage of the experiment by line.

		Diploids			Neotetraploids			Tetraploids		
		D1^a^	D2	DC	N1	N2	NC	T1	T2	TC
Base Population	Maternal Families^b^	113			29			105		
	Individuals^c^	225			57			206		
Selection - One	Fertile Individuals	146			41			195		
	Individuals in Crosses	24	24	24	20		20	24	24	24
	Individuals Common to Control	1	2		12			4	2	
	Seed Families Pairs Created	36	36	36	40		40	36	36	36
Generation One	Maternal Families	34	35	34	30^d^	30^d^	30	30	27	29
	Individuals	134	138	134	109	103	107	107	97	106
Selection - Two	Fertile Individuals	122	138	134	101	101	99	100	95	102
	Individuals in Crosses	24	24	24	24	24	24	24	24	24
	Seed Families Pairs Created	36	36	36	36	36	36	36	36	36
Generation Two	Maternal Families	30	29	33	28	32	30	28	32	30
	Individuals	120	109	126	111	124	112	103	118	115
Selection - Three	Fertile Individuals	114	109	112	108	113	101	103	117	114
	Individuals in Crosses	24	24	24	24	24	24	24	24	24
	Seed Families Pairs Created	36	36	36	36	36	36	36	36	36
Generation Three	Maternal Families	36	34	36	35	35	32	36	36	33
	Individuals	130	128	131	131	132	117	131	129	123
Selection - Four	Fertile Individuals	118	124	130	127	125	110	129	129	123
	Individuals in Crosses	24	24	24	24	24	24	24	24	24
	Seed Families Pairs Created	36	36	36	36	36	36	36	36	36
Generation Four	Maternal Families	15	15	14	15	15	14	15	14	14
	Individuals	30	28	28	30	30	26	30	27	27

a Lines are coded as (D1, D2 = Diploid line 1 and 2, DC = Diploid Control; N1, N2 = Neoautopolyploid line 1 and 2, NC = Neoautopolyploid Control; T1, T2 = Extant Autopolyploid line 1 and 2, TC = Extant Autopolyploid Control).

b Number of maternal families represented within individuals surviving to the end of the generation.

c Number of individuals surviving to the end of the generation.

d Thirty-five seed families were chosen at random from 40 created in first round of selection to found N1 and N2.

Information on the lineage of each plant was used to reduce inbreeding. The inbreeding coefficient for each individual was calculated using Wright's path method using an R program written for this purpose [56].

### Measurement of correlated characters

To determine whether the different ploidy categories showed different correlated responses to selection [36], we examined the phenotypic correlations among, and tested for correlated responses to selection in nine traits: plant height at first flower (distance to top of inflorescence); height of the first flower (from soil); rosette diameter (at 3 weeks); number of leaves produced at first flower; flower size (distance across two opposite petals); style length (base of style to the tip once reflexed); inter-style distance (distance between styles of two adjacent flowers); and leaf length (base to tip). These correlations were calculated using plants from all generations grown in a common environment at the end of the experiment.

We estimated DNA content of plants in the base population and in generation four. We collected young leaves from three plants of each ploidy from the base population and each selection and control line in the fourth generation (N = 36) and used the same flow cytometry protocol as above. The relative 2C DNA content of each plant was estimated by dividing the peak position of *C. angustifolium* by the peak position of *Epilobium hirsutum*, and multiplying the resulting ratio by the 2C DNA content of *E. hirsutum*, 0.87 pg (Kron & Husband, unpublished data). We tested for correlated changes in DNA content because changes in genome size may signal changes in genome structure and can facilitate the evolution of flower timing through changes in cell cycle duration [57].

### Analysis

We estimated evolvability in the three ploidies by estimating realized heritability using 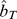, which was calculated as
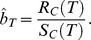


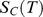
 and 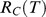, the cumulative directional selection differential and cumulative evolutionary response, respectively, were estimated as,
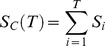
and
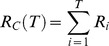
where 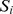 and 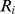 are the directional selection differential and single-generation response for generation i, respectively. The 95% confidence limits were calculated from variances of 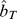 calculated as:
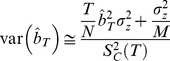


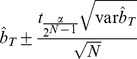
where N is the number of plants grown for each line, M is the number of plants selected included in the selection line, 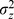 is the base population's phenotypic variance and t is the upper critical value of the t distribution with N-1 degrees of freedom [58]. The coefficient of variation (CV) was calculated as the standard deviation in flowering time for a line divided by the mean flowering time multiplied by 100 giving a measurement of the standing variation for the trait. The confidence interval for the coefficient of variation was calculated using the ci.cv function from the R package MBESS (v3.2.1) [59].

ANOVAs with Tukey's HSD tests were used to test for differences in phenotypic traits among lines, Kruskall-Wallis tests were used to test for differences among lines in inbreeding coefficients and Spearman's rank correlation tests were calculated to investigate phenotypic correlations among traits. In all cases, sterile individuals in each generation were excluded from all calculations. All statistical analyses were performed using R (v 2.12.2) [56].

## Results

**Figure 1:**
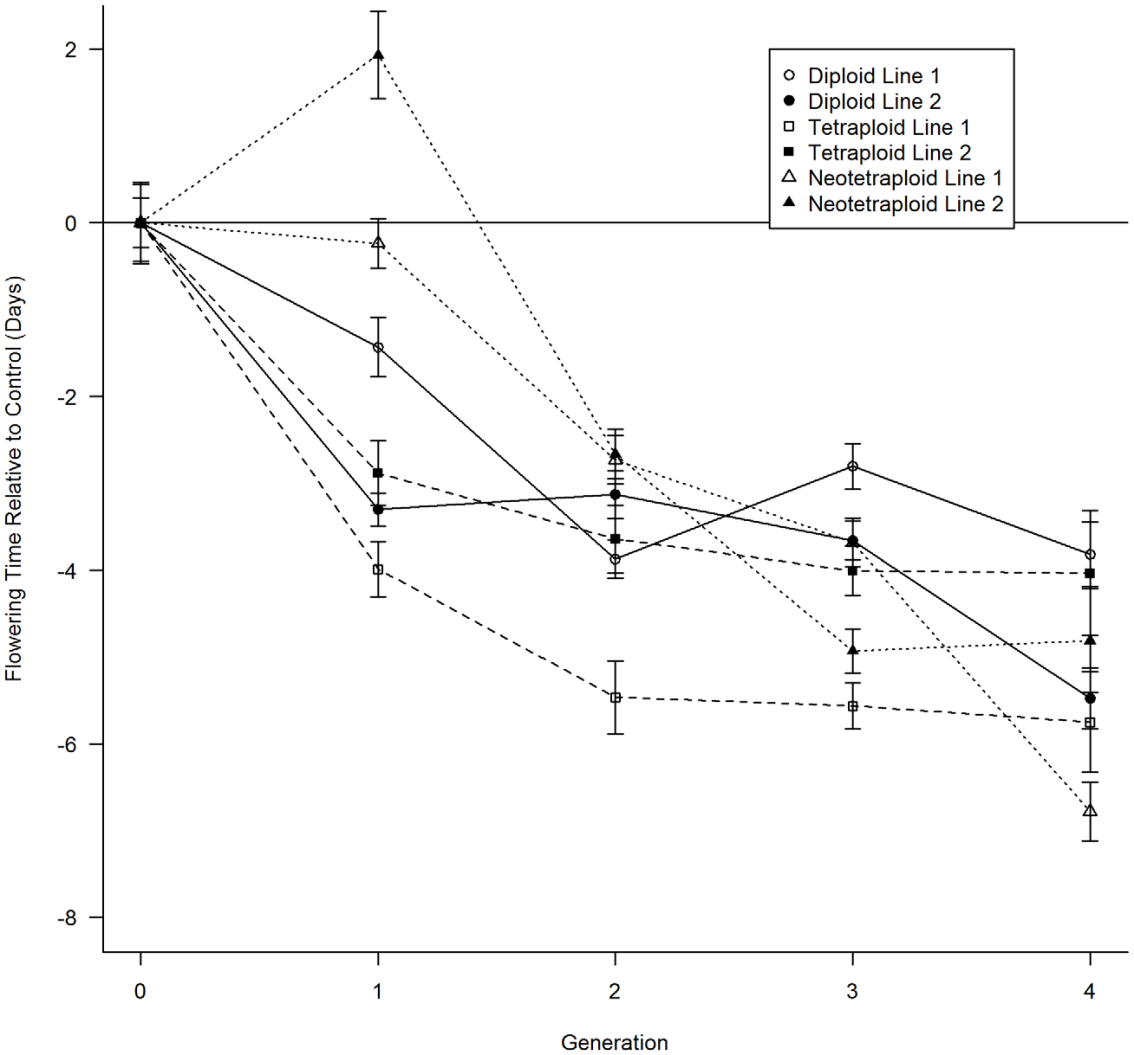
Response to selection for earlier flowering time in diploids, neoautotetaploid and autotetraploid lines. Divergence in flowering time in days relative to the control for diploid, autotetraploid and neoautotetraploid selected lines is shown across the four generations of this experiment.

In the base population, diploids flowered earlier (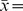
44.2 days from germination to first flower; range = 36 to 58) than tetraploids (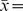
50.3 days; range = 41 to 91), while neotetraploids were intermediate (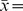
47.1 days; range = 41 to 63) (table 2). After four generations of selection, all selected lines had significantly earlier flowering times compared to unselected control lines. Diploid lines flowered an average of 4.7 (SE = 0.04) days earlier, while neotetraploids and tetraploids advanced flowering by 5.8 (SE = 0.05) days and 4.9 (SE = 0.06) days, respectively (figure 1). For each ploidy, replicate selected lines deviated similarly from controls across the four generations (figure 1). Flowering time in diploid and tetraploid lines declined in the first generation of selection, while neotetraploids had no significant response initially, due in part to lower selection pressure, but responded in subsequent generations.

**Table 2:** Mean phenotypic characteristics of the parental (base) generation with standard errors.

Ploidy state	First Flower		Size at 3 weeks		Height		Leaf size		Flower size		DNA Content		Sterile
	(days)	SE	(mm)	SE	(cm)	SE	(cm)	SE	(mm)	SE	(pg)	SE	(%)
Diploid	44.5^a^	0.2	35.1^a^	0.9	46.3^a^	0.7	18.8^a^	0.2	34.7^a^	0.3	1.46^a^	0.004	35
Neotetraploid	47.4^b^	0.5	46.2^b^	2.2	45.9^a^	1.5	18.9^a^	0.4	33.5^ab^	0.6	2.92^b^	0.004	28
Tetraploid	50.5^c^	0.5	42.6^b^	1.3	58.5^b^	0.9	18.9^a^	0.2	33.3^b^	0.4	2.98^b^	0.004	5

Letters indicate significant differences at p = 0.05 or less between ploidies according to Tukey's HSD.

Averaged across the two selected lines, mean realized heritability differed among ploidy states and had non-overlapping 95% confidence limits. The diploid value (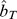 = 0.40; 95% CL 0.38–0.43) was higher than tetraploids (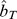 = 0.31; 95% CL 0.28–0.34), suggesting that genome duplication reduces evolvability (table 3). In contrast, neotetraploids (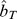 = 0.55; 95% CL 0.51–0.59) had a higher mean realized heritability than either diploids or natural tetraploids, despite having similar variability to diploids in the base population and reduced efficiency of selection due to masking (table 3). The ranking of heritability among ploidy states and the contrast between tetraploids and neotetraploids was consistent among analyses based on sequential comparisons at the end of each generation as well as in the simultaneous comparison of all generations in a common greenhouse environment (results not shown).

**Table 3:** Flowering time in days for each line across generations with realized heritability estimates (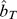) with standard error and 95% confidence limits (standard error = SE, lower limit = LL, upper limit = UL) by line and averaged for each ploidy.

	Diploids			Neotetraploids			Tetraploids		
	Line 1	Line 2	Control	Line 1	Line 2	Control	Line 1	Line 2	Control
Base	44.2^a^			47.1^b^			50.3^c^		
Generation 1	39.1^a^	37.2^b^	40.5^c^	41.0^a^	43.2^b^	41.2^a^	42.9^a^	44.0^a^	46.9^b^
Generation 2	43.8^a^	44.6^a^	47.7^b^	48.3^a^	48.4^a^	51.0^b^	50.3^a^	52.1^b^	55.7^c^
Generation 3	42.5^a^	41.6^a^	45.3^b^	44.4^a^	43.2^b^	48.1^c^	47.0^a^	48.5^b^	52.5^c^
Generation 4	34.3^a^	32.7^b^	38.2^c^	35.3^a^	37.3^a^	42.1^b^	38.4^a^	40.2^a^	44.2^b^
b_T_	0.32	0.49		0.65	0.46		0.37	0.26	
LL	0.30	0.47		0.61	0.42		0.35	0.24	
UL	0.34	0.52		0.69	0.49		0.40	0.28	
SE	0.01	0.01		0.02	0.02		0.02	0.01	
Average b_T_	**0.40**			**0.55**			**0.31**		
LL	0.38			0.51			0.28		
UL	0.43			0.59			0.34		

Letters indicate significant differences at p = <0.05 between ploidies or lines or according to Tukey's HSD.

The inbreeding levels were relatively low in the final generation and Kruskal-Wallis tests indicated that there were no significant differences between lines within ploidy. Specifically, assuming that none of the maternal families were related, that maternal families were fully out-crossed, and that disomic inheritance patterns predominated, the average inbreeding coefficients (F) were: 0.011, 0.029 and 0.028 for the selected diploid lines and control line respectively (K-W 3.55, p = 0.17); 0.035, 0.038 and 0.047 for the selected neotetraploid lines and control line respectively (K-W 2.2, p = 0.33); and 0.017, 0.020 and 0.019 for the selected tetraploid lines and control line respectively (K-W 0.55, p = 0.76). However, the neopolyploids lines had significantly higher levels of inbreeding by the final generation than the diploids or tetraploids (K-W 41.74, p<0.001).

Initially, in the base population, standing variation (CV) for time to first flower was similar in diploid (CV = 7.9) and neotetraploid (CV = 6.4) lines but nearly half that of tetraploid lines (CV = 12.3). This higher variance in tetraploids is consistent with the assumption that initial genetic variability in neopolyploids was similar to the diploids from which they are derived and the expectation that genetic variation in natural populations increases with gene copy number. Standing variation after four generations of selection declined in both selection lines of diploids (−24%, −27%), both tetraploid selection lines (−33%, −24%) and one of the neotetraploid selection lines (−18%) but increased in the second neotetraploid selected line (+37%) compared to the base population. Additionally, a slight increase in CV was seen in the diploid control line (+8%), while a large increase was seen in the neotetraploid control line (123%) (table 4).

**Table 4:** Coefficent of variation in flowering time by generation for each line with 95% confidence intervals.

Line	Base Population	Generation 1	Generation 2	Generation 3	Generation 4
Diploids	7.9 (7.1–8.9)				
Line 1		9.7 (8.6–11.2)	5.4 (4.8–6.2)	6.7 (6.0–7.7)	5.9 (4.7–8.0)
Line 2		6.0 (5.3–6.8)	6.5 (5.7–7.5)	6.0 (5.3–6.9)	5.7 (4.5–7.9)
Controls		7.1 (6.4–8.1)	6.9 (6.1–8.0)	7.2 (6.4–8.2)	8.5 (6.7–11.9)
Neotetraploids	6.4 (5.3–8.2)				
Line 1		7.0 (6.1–8.1)	6.0 (5.3–7.0)	7.1 (6.3–8.1)	5.2 (4.2–7.1)
Line 2		11.3 (9.8–13.2)	6.3 (5.6–7.3)	6.5 (5.8–7.5)	8.8 (7.0–12.0)
Controls		7.9 (7.0–9.2)	10.2 (9.0–11.9)	7.8 (6.9–9.0)	14.2 (11.2–19.8)
Tetraploids	12.3 (11.2–13.7)				
Line 1		7.5 (6.6–8.7)	8.4 (7.4–9.8)	6.5 (5.8–7.4)	8.2 (6.6–11.2)
Line 2		8.3 (7.2–9.7)	8.1 (7.2–9.3)	6.8 (6.0–7.7)	9.3 (7.4–13)
Controls		11.6 (10.2–13.6)	8.1 (7.2–9.3)	6.6 (5.9–7.6)	9.8 (7.8–13.7)

There was no significant change in DNA content per nucleus in any of the selected lines after four generations of selection compared to the respective control lines (diploids: F_2,5 = _1.2, p = 0.74; neotetraploids: F_2,5_ = 7.22, p = 0.07; tetraploids: F_2,5_ = 0.54, p = 0.78) or with plants from the base population (diploids: F_2,5_ = 1.41, p = 0.65; neotetraploids: F_2,5_ = 1.62, p = 0.57; tetraploids: F_2,5_ = 0.09, p = 0.18). Furthermore, the DNA content of neotetraploids and extant tetraploids was not significantly different (F_2,5_ = 0.64, p = 0.43).

**Figure 2:**
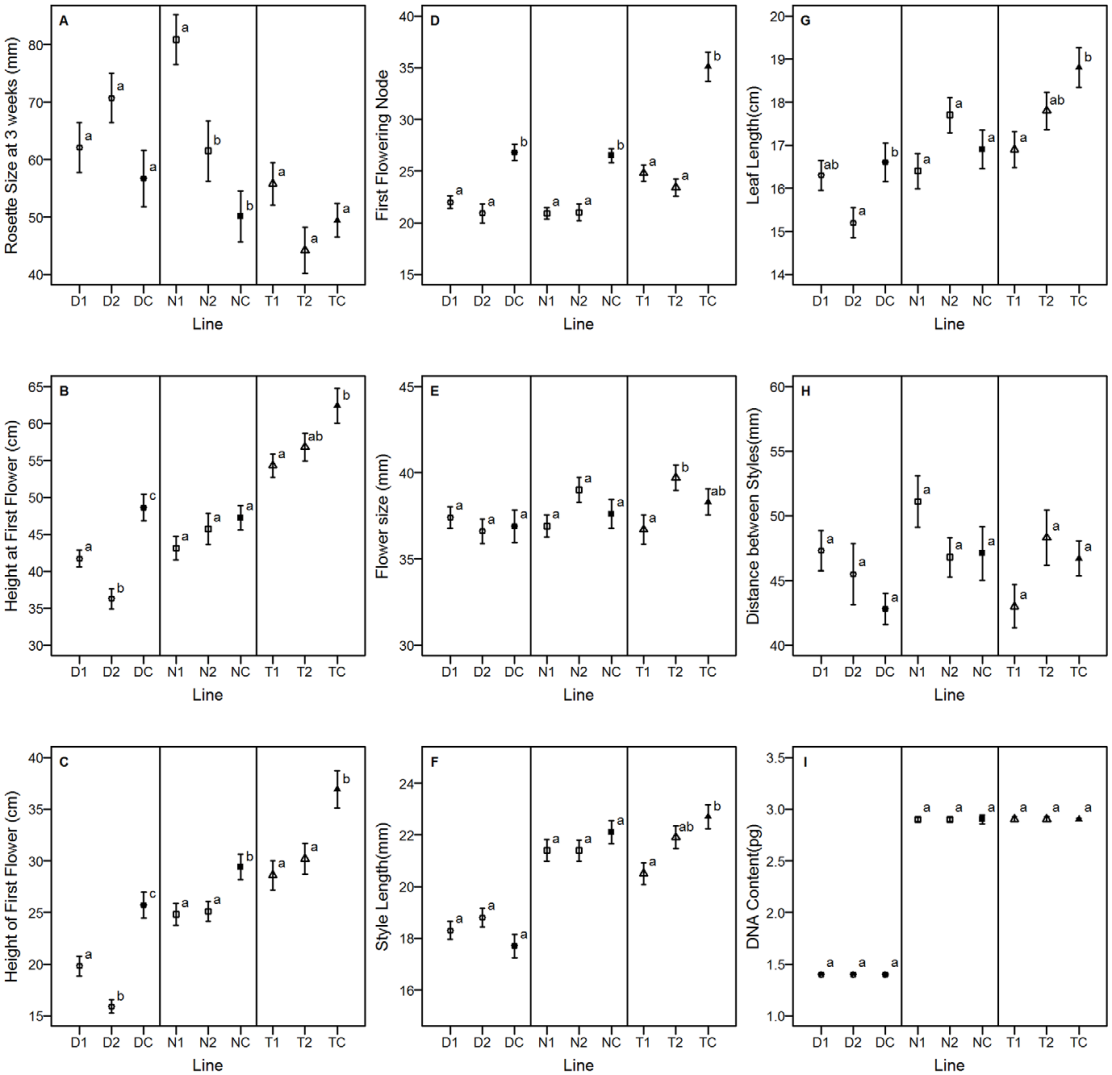
Correlated responses to selection. Correlated responses to selection across lines within ploidies for the fourth generation (D1, D2 = Diploid line 1 and 2, DC = Diploid Control; N1, N2 = Neoautotetraploid line 1 and 2, NC = Neoautotetraploid Control; T1, T2 = Extant Autotetraploid line 1 and 2, TC = Extant Autotetraploid Control). Means with standard error bars are presented with different letters indicating significant differences at the p<0.05 level or less using Tukey's HSD within the ploidy for A) rosette size three weeks after planting (mm), B) total plant height at first flower (cm), C) height of first flower (cm), D) number of nodes produced before the first node with a flower, E) flower size across opposite petals (mm), F) style length from the base of the flower to end of fully reflexed stigma (mm), G) leaf length from stem to tip (cm), H) distance between styles of fully opened adjacent flowers (mm), and I) DNA content as measured by flow cytometry (pg).

Within the final grow out of all generations, plants from the base population and control lines showed phenotypic correlations with time to flower that were similar among ploidies. In all ploidies, time to flower was positively correlated with height at first flower, height of the first flower, average leaf length and number of nodes produced before the first flower. Additionally, time to flower was negatively correlated with rosette size at three weeks. Tetraploids also showed a negative correlation between flower size and days to first flower (table 5). All but three traits, DNA content, flower size, and inter-style distance, had correlated responses to selection in at least one line and ploidy (figure 2).

**Table 5:** Spearman rank correlations between characteristics in the base population.

	Days	Height	F. Ht.	Leaf	Flower	Style	Int	Rosette
Diploids								
Total height	**0.3**							
Height of first flower	**0.3**	**0.73**						
Leaf length	**0.24**	**0.43**	**0.27**					
Flower size	−0.08	**0.46**	**0.38**	**0.45**				
Style length	0	0.11	0.14	**0.28**	**0.5**			
Inter-style distance	−0.09	−0.06	−0.14	0.16	0.07	**0.31**		
Rosette Size at 3 weeks	**−0.73**	**−0.26**	−0.11	**−0.23**	0.03	0.01	0.01	
Nodes to first flower	**0.21**	**0.38**	**0.53**	0.03	0.06	−0.03	−0.16	0.12
Neotetraploids								
Total height	**0.3**							
Height of first flower	**0.5**	**0.66**						
Leaf length	**0.2**	**0.32**	**0.26**					
Flower size	−0.02	0.06	−0.09	−0.14				
Style length	−0.08	0.16	−0.02	−0.14	**0.45**			
Inter-style distance	**−0.3**	**0.22**	−0.04	0.12	0.15	**0.29**		
Rosette Size at 3 weeks	**−0.56**	**−0.26**	−0.16	**−0.22**	−0.1	0.02	0.06	
Nodes to first flower	**0.31**	**0.21**	**0.48**	−0.18	−0.15	0.04	**−0.24**	0.02
Tetraploids								
Total height	**0.59**							
Height of first flower	**0.67**	**0.85**						
Leaf length	**0.47**	**0.38**	**0.36**					
Flower size	**−0.2**	0.01	−0.12	0.06				
Style length	0.02	**0.22**	0.17	0.01	**0.33**			
Inter-style distance	−0.17	0.09	0.06	0.04	0.17	**0.25**		
Rosette Size at 3 weeks	**−0.56**	**−0.27**	−0.24	**−0.2**	0.07	0.07	0.07	
Nodes to first flower	**0.56**	0.5	**0.67**	0.07	−0.15	0.17	−0.09	0

Boldface indicates Spearman's rank correlation coefficients with significance at p = 0.05 or less.

Neotetraploids had three traits that showed positively correlated responses to selection in at least one line: rosette size, number of nodes produced to first flower, and the height of the first flower. This was fewer than either tetraploids (5 correlated traits) or diploids (4 correlated traits). Tetraploids and diploids shared the same correlated traits (plant height at first flower, height of first flower, leaf length, and the number of nodes to first flower), but tetraploids also had a correlated response in style length. Unlike tetraploids, neotetraploids showed no correlated responses in height at first flower, leaf size or style length, despite phenotypic correlations with the first two traits. However, neotetraploids did show a decrease in rosette diameter at three weeks as correlated response to selection.

## Discussion

Experimental evolutionary studies can provide important insights into the genetic and genomic factors influencing rates and directions of adaptation. Here we find that polyploidy has a significant effect on the response to selection for early flowering in *Chamerion angustifolium* (fireweed). Autotetraploid lines derived from natural populations had significantly lower realized heritabilities than those from natural diploid populations, suggesting that the potential to respond to a given selection pressure is reduced by genome duplication. This result is consistent with our prediction that, because of the high genetic variability in this species, the rate of adaptation in will primarily be limited by the rate at which beneficial mutations can spread in a population. This rate can be diminished in polyploids as a result of masking [15]. This result is also consistent with two earlier studies of adaptive responses in yeast, which found that, for large populations, haploids have a greater evolutionary response than diploids [17], [18]. However, this is the first time that the ability of a natural population of polyploids to respond to selection has been directly evaluated in concert their diploid progenitor.

Neotetraploid fireweed had significantly higher realized heritability than either diploid or extant tetraploid fireweed, contrary to our expectations. Based on previous work with fireweed [48], [60], experimental work with yeast [17]–[19], [61], and theoretical work for polyploids [6], we expected neotetraploids to have the lowest adaptive response for two reasons. First, we expected the neotetraploids to have lower genetic variability than diploids or extant autotetraploids because they were synthesized from a small number of parents, and, as they are newly synthesized, they have not had time to accumulate variability through mutation or repeated outcrossing. Second, we expected the neotetraploids, like extant tetraploids, to suffer from reduced efficiency of selection due to increased masking of alleles that contribute to early flowering time. Taken together, these factors should cause reduced evolvability in neotetraploids. Additionally, we would expect that the slightly higher inbreeding in the neotetraploid lines would reduce the response to selection further [48], [60].

We offer three potential explanations for why the neotetraploids showed significantly higher realized heritability than either the diploids or the extant autotetraploids. The first possibility is that greater variability for flowering time was captured in the neotetraploid base population despite the small number of founders, as the original material was derived from different source populations. The neotetraploid base population for this experiment was derived from the progeny of diploid individuals from six locations within the diploid-tetraploid hybrid zone of the Canadian Rocky Mountains (and only five locations were used to establish the control and selection lines). In contrast, seeds used to establish diploid and tetraploid base populations were each drawn from a single location. This may have introduced greater variation for flowering time within the neotetraploid base than was present in the diploid or extant tetraploid lines. However, several lines of evidence suggest that this is unlikely. First, based on previous research, diploid plants from the source populations can be considered part of one panmictic population as fireweed is highly outcrossing with strong seed dispersal, and populations show little genetic differentiation or local adaptation [45], [46], [50], [51]. This reduces the probability that sampling additional populations resulted in capturing a greater diversity of flowering time alleles. Second, the neotetraploids that contributed to the base population represent the offspring of 55 individuals that successfully transformed – a much narrower genetic base than that represented by the diploid and tetraploid lines, which were each drawn from over 100 outcrossed maternal families. Finally, the coefficient of variation for flowering time in the base population of the neotetraploids (6.4) was lower than that for the diploids (7.9) and preliminary genetic work on the selection lines using AFLPs indicate that the effective number of alleles and expected heterozygosity of the parental neotetraploid and diploid groups are very similar (Husband and Martin unpublished data). Each of these lines of evidence suggests that the response seen in the neotetraploids is unlikely to be the result of greater variation in the base population.

A second explanation for high evolvability of neotetraploids is that genome duplication resulted in a release of variability similar to that seen in allopolyploids. Allopolyploid *Brassica napus* resynthesized from *B. rapa* and *B. oleracea* have been found to exhibit extensive *de novo* variation for life-history traits including flowering time [25], [29]. Similar mechanisms may be acting in fireweed following autopolyploidization. A line of evidence supporting this possibility is that the coefficient of variation in flowering time increased in one of the neotetraploid selection lines by 38% and in the neotetraploid control line by 123% from the base generation to the final selected generation, whereas the CV in diploids and tetraploids selected lines showed decreases averaging 25% and 27% respectively. These striking trends are not a product of having greater maternal effects in the diploid and tetraploid lines, which were derived directly from field collected seed. If this were the case, the diploid control line would have shown stronger reductions in CV over time. Instead, the line exhibited a weak increase in CV, while neotetraploid show a marked increase. Previous work on resynthesized allopolyploids suggests that increased phenotypic variation may be the result of chromosomal rearrangement or changes in epigenetic processes following polyploidization [21]–[30]. These changes, which have been interpreted as an explanation for the success of polyploids, have been primarily attributed to the hybridity of the allopolyploid genomes, however our results suggest that genome duplication *per se* may also play a role in inducing these changes.

A final explanation for the greater than expected ability to respond to selection in the neotetraploids could be altered patterns of chromosome pairing during meosis. For example, the efficiency of selection may differ for tetraploids and neotetraploids if meiotic pairing and segregation change with age. Cytological studies of extant fireweed found 35% of chromosomes formed quadrivalents at meiosis [47]. However, neotetraploids may form quadrivalents at higher rates, since selection has had little opportunity to reduce meiotic abnormalities [62]. This chromosomal behaviour may contribute to increased efficiency of selection in the neotetraploid lines as the conditions for the spread of partial recessive mutations are less stringent with tetrasomic inheritance than with disomic inheritance [6].

We explored two genomic features potentially associated with the rapid evolutionary response in neotetraploids: genome downsizing and relaxed trait correlational structure. We found no evidence that genome size changed in any ploidy during this experiment. As a result, the increased evolvability in neotetraploids was associated with a decrease in genome size [57], [63]. While it might be expected that the response to selection could be enhanced in polyploids if increased gene copy number results in weaker genetic correlations [36] through subfunctionalization and neofunctionalization, there is no evidence that neotetraploids had fewer phenotypic correlations or fewer correlated responses to selection than diploids. However, the neotetraploids did show fewer correlated responses to selection than tetraploids. This finding is qualitatively similar to Oswald and Nuismer's finding that although autopolyploidization resulted in immediate phenotypic differentiation from diploids, it did not alter the genetic covariance matrix of neoautopolyploid *Heuchera grossularifolia* [36].

Age-dependent effects of genome duplication have important implications for the process of polyploid evolution. We find that neotetraploids have phenotypes that are similar to diploids (plant height at first flower), and intermediate in value (time to flower) or comparable to tetraploids (rosette size at three weeks). This suggests that neotetraploid fireweed may not have fully resembled the naturally occurring tetraploids when they first originated, and therefore, may not have been initially reproductively or ecologically divergent [49], [64]. This may be common, as recent work with neohexaploids of *Achillea borealis* indicates that approximately a third of the fitness difference between extant tetraploids and extant hexaploids in dune habitats is the result of genome duplication *per se* [65]. If novel tetraploids initially resemble their diploid progenitors, then the selective forces promoting their exclusion and preventing persistence may be strong [8], [66]–[68]. The chance that a polyploid will persist may then depend on rapidly diverging from diploids. Our evidence suggests that this chance may be enhanced by the ability of neotetraploids to respond more quickly to selection than extant diploids or tetraploids. However, additional research in this system as well as in other systems is needed to determine whether the pattern observed here is typical for other traits or other species and to continue disentangling the role of genome duplication and hybridization in the evolutionary success of polyploids.

Here we provide novel insights into the fundamental evolutionary question, to what extent adaptive divergence is limited by the structure of the genome [6]. We show experimentally that in fireweed the rate of evolutionary response is affected by both genome copy number and time since genome duplication. Response in naturally occurring autotetraploids was 20% lower than in co-occurring diploids, indicating that adaptation is likely limited by the rate at which beneficial alleles can spread through the populations in tetraploids [6]. In contrast, the 37% increase in evolutionary response seen in newly synthesized autotetraploids compared to diploids suggests that whole genome duplication without hybridization may alter adaptation rate. This suggests that new polyploids may have more dynamic genomes than older polyploids regardless of mode of origin and opens an interesting line of inquiry as we develop our understanding of the prevalence of polyploidy throughout the eukaryotes.
